# Analyzing attempt and success factors for amputated digit replantation in Japan using the diagnosis procedure combination database

**DOI:** 10.1038/s41598-024-62879-2

**Published:** 2024-05-28

**Authors:** Daishi Hamada, Hitoshi Suzuki, Keiji Muramatsu, Yukichi Zenke, Makoto Kawasaki, Kiyohide Fushimi, Shinya Matsuda, Akinori Sakai

**Affiliations:** 1 Trauma Reconstruction Center, School of Medicine, University of Occupational and Environmental Health, 1-1 Iseigaoka, Yahatanishi-Ku, Kitakyushu, 807-8555 Japan; 2 Department of Orthopedic Surgery, School of Medicine, University of Occupational and Environmental Health, 1-1 Iseigaoka, Yahatanishi-Ku, Kitakyushu, 807-8555 Japan; 3 Department of Preventive Medicine and Community Health, School of Medicine, University of Occupational and Environmental Health, 1-1, Iseigaoka, Yahatanishi, Kitakyushu, 8078555 Japan; 4 Department of Health Policy and Informatics, Tokyo Medical and Dental University Graduate School, Yushima, Bunkyo-Ku, Tokyo, 113-8510 Japan

**Keywords:** Replantation, Amputation, Success factor, Japanese trends, Medical research, Risk factors

## Abstract

The number of amputated finger replantation has declined in the USA and Germany in recent years; however, there have been no reports on recent trends in Japan. We examined the current practices, attempts, and success factors of digit replantation in Japan. We hypothesized that the rates of digit replantation and success rates were consistently standardized in Japan. The diagnosis procedure combination database was used to analyze 14004 cases from April 2014 to March 2020, excluding multiple-digit amputations, thus focusing on 13484 patients. We evaluated replantation success rates and identified factors influencing replantation decisions using multiple logistic regression analysis. The key findings included a higher frequency of replantation in thumb cases and surgeries during overtime hours, on Sundays, and in educational institutions. Success rates were notably higher for thumb replantations and patients under 20 years of age. Patients over 65 years of age treated with urokinase showed higher failure rates, unrelated to regional or hospital case volumes. The number of amputated digit replantation surgeries in Japan was high during overtime hours, on Sundays, and in educational institutions. Region, hospital type, and hospital case volume were not associated with a low success rate across Japan.

## Introduction

Thumb replantation was initially successfully performed by Komatsu and Tamai^1^ in 1963 and has since become a widely adopted procedure globally that yields favorable results^1^. Failure after digit replantation significantly affects a patient’s activities of daily living. The reported success rates for the replantation of amputated digits range between 66.3% and 91.4%^2^. The implementation and success rates of digit replantation are very crucial because successful replantation is associated with better pain control and function than those with amputation^3^.

Traumatic digit amputation predominantly affects young working-age male individuals, with an increasing number of replantation procedures observed in Asian countries^4–6^. In contrast, the United States and Germany have seen a decline in the replantation rates owing to a refinement of indications, fewer patients with amputations, and declining reimbursement^6–8^. A difference in success rates was reported between facilities where many surgeries were performed and those in which a small number of surgeries were performed. If the number of surgeries continues to decline, the concern is that the technique of replantation will become more dispersed, and the success rate of surgeries will decline^8–12^.

Although some epidemiological studies have reported on the number of replantation procedures and treatment outcomes in the United States and Asian countries^4–6^, large-scale epidemiological studies specific to Japan are lacking, with most studies consisting of small case series^13^. Moreover, there are few reports comparing the characteristics of patients in whom replantation was performed and patients in whom amputation was performed. Thus, in this study, we aimed to identify the factors influencing the selection of digit replantation surgery in Japan and to examine the subsequent success or failure of replantation procedures. We hypothesized that the rate and success rate of amputated digit replantation in Japan would be standardized with no regional differences. Furthermore, we hypothesized that the rate of replantation would vary depending on the day of the week and time of the day of the injury.

## Methods

### Data sources

We used data registered in the diagnosis procedure combination (DPC) inpatient database from April 2014 to March 2020 in Japan. The database contains comprehensive medical information, including data on surgical procedures and medications indexed in the original Japanese codes assigned by the Ministry of Health, Labour, and Welfare of Japan (Tokyo, Japan). Data were collected by the DPC Research Institute (a nonprofit organization) in collaboration with the Ministry of Health, Labour, and Welfare of Japan. During the study period, the DPC Research Institute collected data from 1478 hospitals that provided their DPC data to the database for research purposes. This study adhered to the principles of the Declaration of Helsinki and its amendments. The requirement for informed consent was waived owing to the retrospective nature of the study.

### Ethics approval

The authors conducted the present study in accordance with the “Ethical Guidelines for Life Science and Medical Research Involving Human Subjects” issued by the Ministry of Education, Culture, Sports, Science, and Technology, the Ministry of Health, Labor, and Welfare, and the Ministry of Economy, Trade, and Industry. The study protocol was approved by the Ethics Review Committee for Clinical Research of the University of Occupational and Environmental Health Institutional Review Board (Approval No. R1-067). The requirement for informed consent was waived by the Ethics Review Committee for Clinical Research of the University of Occupational and Environmental Health Institutional Review Board owing to the retrospective nature of the study.

### Data collection

Patient data labeled with codes S680 (thumb amputation), S681 (amputation of digits other than the thumb), and S682 (multiple-digit amputation), based on the International Classification of Diseases 10th revision, were extracted from the DPC database. Patients with multiple-digit amputations were excluded from the logistic regression analysis of replantation rates. Moreover, cases of devascularization (S654, S655) were excluded to ensure uniformity of severity. The success and failure rates are described below. A total of 13484 patients were enrolled in this study. Epidemiological data on reimplanted amputated digits were collected, including the injured digit, patient’s age, day of the week on which surgery was performed, and whether it was an overtime-hour operation.

### Factor setting

The factors that influenced the decision to perform replantation in cases of digit amputation were identified. These factors included age, injured digit, comorbidities at admission (such as diabetes, heart failure, and cerebrovascular accidents), day of the week on which surgery was performed, operation start time, and hospital classification (educational or non-educational institution). We also investigated factors associated with success/failure after digit replantation, hospital case volume, and type of intravenous anticoagulation used (prostaglandin E1, heparinoid, and urokinase). The rate of attempted replantation was defined as the percentage of patients who underwent replantation surgery for amputated digits. With reference to previous reports, failure was defined as cases in which a patient had undergone flap surgery, amputation, free flap transfer, wound treatment, or local negative pressure closure procedures within 2 weeks after digit replantation surgery during the same hospitalization period^4,12,14,15^. Multiple-digit amputation cases were excluded from the logistic regression analysis owing to the difficulty in calculating the failure rate when including them.

### Statistical analysis

Factors influencing the decision to perform replantation for amputated digits and those influencing success/failure after replantation were analyzed using logistic regression analysis with adjusted models. All calculations were conducted using STATA ver. 16.1 (Stata, College Station, TX, USA). Significance was set at *P* < 0.05 for all analyses. Data are presented as mean ± standard deviation or as odds ratios (ORs) with 95% confidence intervals (CIs), unless otherwise stated.

### Conference presentation

Oral presentation in part at the 12th Congress of the World Society for Reconstructive Microsurgery, in Singapore, August 17 through 19, 2023.

## Results

### Characteristics of digit amputations in Japan

The injured digits included 2,086 thumbs (14.9%) and 11,398 fingers (81.4%). Patients were aged 0–19 years in 925 (6.9%) cases of injured digits, 20–64 years in 9,382 (69.6%) cases, and > 65 years in 3,177 (23.6%) cases. Analysis of the operation start time determined that 11,895 (88.2%) patients underwent surgery during the day, and 1,589 (11.8%) patients underwent surgery in the afternoon (after 5:00 PM) Urokinase was administered in 874 of 13,484 (6.5%) patients and not administered in 12,610 (93.5%) (Table 1).

**Table 1:** Characteristics of digit amputations in Japan.

Injured digit	Thumb	2086 (14.9%)
	Others	11398 (81.4%)
	Multi-amputated	520 (3.7%)
Age (y)	0–19	925 (6.9%)
	20–64	9382 (69.6%)
	65+	3177 (23.6%)
Hospital arrival time	Working hours	11895/13484 (88.2%)
	After working hours	1589/13484 (11.8%)
Administration of urokinase	Yes	874/13484(6.5%)
	No	12610/13484(93.5%)

### Replantation surgery rates after digit amputation

Replantation was performed in 1,786 (13.2%) of the 13,484 patients. There was a significantly higher rate of patients aged < 19 years (OR 1.25; 95% CI 1.02–1.55; *P* = 0.03) and > 65 years (OR 1.75; 95% CI 1.54–1.99; *P* < 0.001) who underwent replantation than of those aged 20–64 years. Other factors that led to a higher digit replantation rate included thumb injury (OR 1.25; 95% CI 1.07–1.46; *P* = 0.004), presence of diabetes (OR 1.58; 95% CI 1.27–1.96; *P* < 0.001), Sunday as the day of surgery (OR 2.84; 95% CI 2.28–3.52; *P* < 0.001), surgery performed during overtime hours (OR 5.26; 95% CI 4.53–6.10; *P* < 0.001), and educational institution (OR 9.21; 95% CI 6.92–12.27; *P* < 0.001). In non-educational institutions, 1378 (11.1%) of 12,367 cases underwent replantation, while in educational institutions, 408 (36.5%) of 1117 cases underwent replantation. No regional differences were found in the digit replantation surgery rates (Table 2).

**Table 2:** Odds ratios for replantation attempts.

Variable		Univariate			Multivariate		
		Odds ratio	95% CI	P-value	Odds ratio	95% CI	P-value
Age (y)	0–19	2.03	1.67–2.45	< 0.001	1.25	1.02–1.55	0.03
	20–64		1 [reference]			1 [reference]	
	65 +	2.67	2.37–3.00	< 0.001	1.75	1.54–1.99	< 0.001
Injured digit	Thumb	1.27	1.11–1.46	< 0.001	1.25	1.07–1.46	0.004
	Others		1 [reference]			1 [reference]	
Comorbidity	Diabetes	2.12	1.74–2.58	< 0.001	1.58	1.27–1.96	< 0.001
	Heart failure	2.18	1.53–3.1	< 0.001	1.4	0.95–2.05	0.09
	Cerebrovascular accident	2.51	1.48–4.24	< 0.001	1.61	0.92–2.80	0.1
Day of the week	Sun	2.51	2.05–3.07	< 0.001	2.84	2.28–3.52	< 0.001
	Mon		1 [reference]			1 [reference]	
	Tue	1.05	0.87–1.28	0.61	1.08	0.88–1.33	0.45
	Wed	0.97	0.8–1.18	0.79	0.94	0.76–1.16	0.54
	Thu	1.02	0.84–1.24	0.81	1.02	0.83–1.25	0.87
	Fri	1.15	1.45–2.16	0.15	1.05	0.85–1.29	0.65
	Sat	1.77	0.08–0.11	< 0.001	0.87	0.69–1.09	0.22
Operation start time	Overtime	6.02	5.29–6.85	< 0.001	5.26	4.53–6.10	< 0.001
Local area	Hokkaido	0.62	0.36–1.08	0.09	0.61	0.33–1.15	0.13
	Tohoku	1.13	0.74–1.71	0.56	1.06	0.66–1.71	0.8
	Kanto		1 [reference]			1 [reference]	
	Chuubu	0.76	0.54–1.05	0.1	0.76	0.52–1.11	0.16
	Kinki	0.97	0.7–1.35	0.86	1.17	0.79–1.71	0.43
	Chuugoku	0.85	0.55–1.31	0.46	0.8	0.49–1.32	0.38
	Shikoku	1.05	0.61–1.82	0.85	0.93	0.50–1.76	0.83
	Kyuushuu	0.76	0.54–1.07	0.12	0.77	0.52–1.14	0.19
Hospital status	Educational	19.23	14.4–25.67	< 0.001	9.21	6.92–12.27	< 0.001

### Success/failure rates after replantation surgery

Among the 1,786 patients who underwent replantation, 169 underwent additional surgery for necrosis and/or other complications within 2 weeks after surgery (failure), and the failure rate after replantation was 9.5%. Factors affecting failure after replantation included age > 65 years (OR 1.53; 95% CI 1.08–2.15; *P* = 0.016) and urokinase use for antithrombotic therapy (OR 1.47; 95% CI 1.03–2.08; *P* = 0.03). Although the replantation attempt rate was higher among patients with diabetes, the subsequent success rate did not differ from that in patients without diabetes (OR 1.06; 95% CI 0.60–1.87; *P* = 0.84). The success rate of replantation surgery was higher for the thumb (OR 0.32; 95% CI 0.18–0.57; *P* < 0.001) and for surgeries performed in the Kinki district (OR 0.46; 95% CI 0.26–0.83; *P* = 0.01). No significant differences were observed in the failure rates based on the hospital case volume, time of the day, or day of the week. Moreover, no significant difference was observed between education and non-education hospitals (Table 3).

**Table 3:** Odds ratios for failure after replantation.

Variable		Univariate			Multivariate		
		Odds ratio	95% CI	P-value	Odds ratio	95% CI	P-value
Age (y)	0–19	0.63	0.32–1.24	0.18	0.59	0.29–1.18	0.14
	20–64		1 [reference]			1 [reference]	
	65 −	1.31	0.95–1.83	0.1	1.53	1.08–2.15	0.016
Injured digits	Thumb	0.34	0.19–0.61	< 0.001	0.32	0.18–0.57	< 0.001
	Others		1 [reference]			1 [reference]	
Comorbidity	Diabetes	1.01	0.59–1.74	0.97	1.06	0.60–1.87	0.84
	Heart failure	0.38	0.09–1.58	0.18	0.36	0.084–1.52	0.16
	Cerebrovascular accident	0.41	0.06–3.08	0.39	0.36	0.047–2.71	0.32
	Cancer	0.87	0.11–6.82	0.9	1.15	0.14–9.53	0.9
Day of the week	Sun	1	0.57–1.77	1	1.13	0.62–2.06	0.68
	Mon		1 [reference]			1 [reference]	
	Tue	1.16	0.66–2.04	0.6	1.19	0.67–2.12	0.56
	Wed	0.54	0.27–1.05	0.07	0.55	0.27–1.09	0.09
	Thu	0.67	0.36–1.25	0.21	0.62	0.33–1.19	0.15
	Fri	0.74	0.4–1.37	0.34	0.72	0.39–1.35	0.31
	Sat	1.38	0.8–2.37	0.25	1.23	0.68–2.21	0.49
Operation start time	Overtime				1.22	0.83–1.80	0.32
Local district	Hokkaido	0.63	0.22–1.83	0.4	0.54	0.18–1.62	0.27
	Tohoku	1.55	0.89–2.7	0.12	1.25	0.69–2.25	0.46
	Kanto		1 [reference]			1 [reference]	
	Chuubu	1.22	0.74–2	0.43	1.07	0.63–1.80	0.81
	Kinki	0.55	0.31–0.96	0.04	0.46	0.26–0.83	0.01
	Chuugoku	1.03	0.53–1.99	0.93	0.78	0.39–1.57	0.48
	Shikoku	1.3	0.64–2.62	0.48	1.16	0.55–2.45	0.7
	Kyuushuu	1.02	0.62–1.7	0.93	0.92	0.55–1.56	0.77
Hospital status	Educational	1.05	0.72–1.54	0.81	1.01	0.68–1.52	0.95
Hospital case volume	1–4 cases		1 [reference]			1 [reference]	
	5–9 cases	0.95	0.58–1.57	0.85	0.91	0.55–1.53	0.73
	10–14 cases	1.48	0.9–2.43	0.12	1.6	0.95–2.69	0.08
	15–19 cases	0.93	0.49–1.76	0.82	0.84	0.42–1.67	0.62
	≥ 20 cases	0.99	0.61–1.62	0.97	1.05	0.62–1.75	0.86
Intravenous drip	Heparinoid	1.15	0.54–2.42	0.72	1.09	0.50–2.38	0.84
	PGE1	1.06	0.74–1.53	0.74	0.85	0.57–1.27	0.44
	Urokinase	1.55	1.11–2.15	0.01	1.47	1.03–2.08	0.03

## Discussion

The factors that contributed to the replantation rate were age < 19 years, age > 65 years, thumb amputation, concomitant diabetes, surgery on Sunday/during overtime, and educational institutions. The factors affecting the postoperative failure rate were age > 65 years and urokinase use. However, the replantation success rate was influenced by thumb amputation in Kinki.

The rate of performing replantation for amputated digits in this study was 13.2%, which was close to the rates of 18 and 11.2% reported by Brown et al.^9^ and Shale et al.^14^, respectively, in the United States. The failure rate of replantation in this study was 9.5%, which was similar to 8.4% reported in Taiwan^4^. The high frequency of replantation for thumb amputation and the similarly high frequency of replantation in younger patients were consistent with those in previous studies^16^^.^ These data provide a comprehensive overview of digit replantation in Japan.

The higher rate of attempted replantation in elderly patients was unexpected with respect to the replantation rates. It is possible that patients aged 65 years or older were more willing to undergo replantation (even if the treatment time was long) than the working-age group, aged 20–64 years, who sought to return to work as soon as possible after injury. This is the first study to show that finger replantation is more frequently selected in surgeries for finger amputations beyond normal working hours, such as on Sundays and after work hours.

This study also showed that the replantation rate was high in educational institutions, which was comparable to previous reports^12,15^. Cho et al. reported that replantation was 2.4 times more common in educational hospitals located in urban centers than in other areas^12^. As 36.5% of digit amputation cases were replanted in educational hospitals and the failure rate was comparable to that in non-educational hospitals, educational hospitals may play an important role in digit amputation cases.

The success rate of replantation was 84.9% in 2013^14^, while it was 83.4% in 2018^12^. The success rate of replantation in this study was 90.54%, which is comparable with or better than previous results^17–19^. Age affects the failure rate after replantation^17,18^. The present study corroborated this finding by revealing a higher failure rate in patients over 65 years of age. Hospitals with a larger number of cases have lower failure rates after replantation^20^. However, the number of cases at each hospital had no effect on the success rate in this study. Furthermore, the success rate of replantation techniques was higher in the Kinki region than in other regions of the country. A possible reason for the better results in this area than in other areas is the presence of a facility with which Komatsu and Tamai were affiliated. However, this study indicated that the hospital case volume does not affect the success rate of replantation in Japan. These results indicate that replantation for amputated digits is performed in Japan without regional differences, and that the failure rate in all regions is comparable to that in other countries.

Although the rate of replantation was higher among patients with diabetes, the postoperative failure rate was comparable to that among patients without diabetes. We considered that patients with diabetes have a higher rate of reimplantation because of the higher prevalence of diabetes in the elderly population. Our finding that diabetes is not associated with postoperative failure also has implications for surgical indications. Furthermore, antithrombotic therapy after replantation is widely used, and its effectiveness is based on data from studies in rat models^21,22^. Many negative reports exist on the efficacy of antithrombotic therapy after replantation. In this study, the use of heparinoids and PGE1 did not affect the postoperative failure rate^23,24^. However, this study showed that urokinase use was significantly correlated with failure, and it is possible that patients who receive antithrombotic therapy may be severely injured at the time of injury. In fact, urokinase was used in only 6.5% of all cases, and its use may have been preferred in cases with a high probability of failure. Therefore, the risk of failure was not increased by the use of urokinase, but rather reflected the initial severity of the disease.

This study had several limitations. It was a retrospective observational study based on DPC data; therefore, the surgical details are unknown. Second, this study was limited to cases involving hospitals participating in the DPC. Therefore, it does not reflect the results of other institutions. Third, the detailed amputation levels and injury status at the time of amputation were unknown; therefore, this study did not standardize the cases. In particular, the amputation level and injury status are reported to affect the success rate after replantation of amputated digits^25^. However, these data are not standardized and may not reflect the appropriate postoperative outcomes. A further consideration is the definition of failure rate. We defined failure as a case in which the patient underwent reoperation during the same hospitalization within 2 weeks of the initial surgery. Because we could not obtain data on cases in which the patient was readmitted to the hospital and underwent surgery after being discharge, it is possible that we may have underestimated the number of failed cases. Therefore, we were unable to completely capture the actual status of case success/failure.

In the future, standardizing the treatment of amputated digits will be necessary by compiling a database of cases, including the amputation site and the degree of injury. This information will lead to stricter indications for the replantation of amputated digits that should increase the success rate of surgery.

## Conclusion

In Japan, replantation is frequently performed for amputations in young and older adult patients, on the thumbs, in the after hours, on Sundays, and in educational institutions. The success rate is higher for the thumb and lower for older patients and patients using urokinase. Region, hospital type, and hospital case volume were not associated with a low success rate across Japan.

## Data Availability

The datasets generated and/or analyzed during the current study are not publicly available for personal information protection but are available from the corresponding author upon reasonable request. The other contact points are Office of Center for Clinical Research Promotion, Division of Clinical Research, the University of Occupational and Environmental Health (E-mail: rinshokenkyu@mbox.clnc.uoeh-u.ac.jp Tel: +81-93-691-7127) and the Ethics Committee, Graduate School of Medicine, the University of Occupational and Environmental Health.
